# International Health Work

**Published:** 1920-03-27

**Authors:** 


THE PUBLIC WELL-BEING—(*continued*).

### INTERNATIONAL HEALTH WORK.

### Big Programme for Brussels Congress.

THE subject of public health, from the international standpoint, is receiving much wider attention now than ever before. Reference has already been made to the steps taken by, and obligations imposed upon, the League of Nations on health questions, and Sir William J. Collins contributes this month to the journal of the League of Nations Union an interesting summary of the kind of things with which the League seems enabled to deal. He says that there are wide possibilities for advancement in public health questions which have now been opened up by envisaging them from that world-wide standpoint which it is hoped the League of Nations will be in a position to take up successfully.

He takes as one of his illustrations the obligation to endeavour to take steps in matters of international concern for the prevention and control of disease. Epidemic sickness, he says, laughs at territorial frontiers, whether it be Asiatic cholera, Spanish influenza, Levantine plague, the German measles, or the French disease—sorry nomenclature whereby a short-sighted epidemiology seeks to shirk responsibility and foist upon another people the causation of epidemics for which home neglect has too often provided a breeding-ground. The need is now self-evident for world-wide effort to cope successfully with world-wide sources of disease and death. The typhus epidemic still raging in and around Galicia calls for prompt intervention. Early international notification of outbreaks of epidemic or infectious disease, and better sanitary supervision and disinfection of ships and ports, with a view to the localisation and suppression of such outbreaks, will achieve more than the old quarantine system, which often hampered trade without checking disease. Apart from this, Sir William Collins also points briefly to the various other duties which seem to come within the purlieu of the League, among them the promotion of the physical welfare of children and mothers and of general hygienic reforms. He also foreshadows that attention will be directed to the physical and moral ills associated with the drink traffic among native races.

### THE BRUSSELS CONGRESS.

Another indication of growing international concern is the International Congress, organised by the Royal Institute of Public Health. It is to meet in Brussels in May. The British, American, French, and Belgian Governments are giving their approval and support, and amongst the subjects dealt with are State medicine, naval, military, and tropical questions, municipal hygiene, industrial hygiene, women's work, and bacteriology and chemistry. Several of these subjects, of course, are technical and scientific in character, but a great many are of wide social interest and apply very closely to problems of the day. In the section of State medicine, for instance, five papers are specially devoted to child life, while many of the others are directly or indirectly concerned with children, and the women's work section has a series of papers, every one of which concerns children's welfare in some form or other. There are to be nine discussions on different phases, of the problem of venereal disease, the problem of milk supply, the control of tuberculosis, the development of various occupational diseases, and the general question of industrial welfare—these are all down for full discussion. This is a very big field, and it is hoped that the Congress will be able to secure a wide publicity for the most valuable public sections of its work, presented in such a form that they will interest the general reader.

There is virtually nothing in the programme which is not of great public interest and importance, and there is a very distinguished list of experts taking part. There is not room here to give the subjects in detail because the ground to be covered is very comprehensive. One section, however, which is of special interest is that on hygiene and women's work, and the Congress will have the advantage of hearing women discuss the problems involved. Mrs. Mary Scharlieb and Dr. D. Gilbert are the presidents, and there are at least fifteen out of about twenty vice-presidents who are women. The papers include:—

"Women's Work under the Ministry of Health," by Viscountess Rhondda.

"Alcohol as it Affects the Community," by Viscountess Astor, M.P.

"Ante-Natal Care," by Lady Barrett, M.D.

"The Social Life of the Child," by Lady Leslie Mackenzie.

"The Pre-Maternity Ward in Hospitals," by Dr. John W. Ballantyne.

"The Physiology of Childhood," by Miss Winnifred Cullis, D.Sc.

"The Training of Personnel in Maternity and Child Welfare Work," by Mr. J. S. Fairbairn, F.R.C.S.

"Women's Employment and Capacity for Motherhood," by Miss Letitia Fairfield, M.D.

"Mothercraft from Mothers' Point of View," by Mrs. H. B. Irving.

"Ante-Natal Work and the Prevention of Infant Mortality," by Bt.-Col. J. R. Kaye, Medical Officer of Health for the West Riding of Yorkshire.

"The Co-ordination of the Various Branches of a Child Welfare Centre," by Miss Christine Murrell, M.D.

"Duties and Status of Midwives," by Miss Rosalind Paget, Member of Central Midwives Board.

"Housing and Infant Mortality," by Dr. S. G. Moore, Medical Officer of Health for Huddersfield.

"Institutional Management of Young Infants," by Dr. Eric L. Pritchard.

"Birth Rate and Empire," by Dr. C. W. Saleeby.

"The Hygienic Value of the Village Institute in Denmark," by Miss Smith Rossie.

